# Supplementary material for: Vertical eddy iron fluxes support primary production in the open Southern Ocean
Source: Nat Commun. 2020 Feb 28;11:1125. doi: 10.1038/s41467-020-14955-0 (PMC7048949; doi:10.1038/s41467-020-14955-0)
Supplement: Supplementary file 1 — Supplementary Information [file 41467_2020_14955_MOESM1_ESM.pdf]

Vertical Eddy Iron Fluxes Support Primary Production in the Open Southern Ocean

Uchida et al.

Nature Communications

Supplementary Information

Vertical Eddy Iron Fluxes Support Primary Production in the Open Southern Ocean

Takaya Uchida[1], Dhruv Balwada[2], Ryan Abernathey[1,3], Galen McKinley[1,3], Shafer Smith[2] & Marina Lévy[4]

[1]Department of Earth and Environmental Sciences, Columbia University in the City of New York
[2]Center for Atmosphere Ocean Science, Courant Institute of Mathematical Sciences, New York University
[3]Division of Ocean and Climate Physics, Lamont-Doherty Earth Observatory
[4]Laboratoire d'Océanographie et du Climat, Institut Pierre Simon-Laplace

Content of this file

Supplementary Figures (Supplementary Figure 1, 2,…, 8);
Supplementary Tables (Supplementary Table 1, 2);
Supplementary Notes (Supplementary Note 1, 2,…, 7);
Supplementary References.

Introduction

Here, we provide some details on the model configuration in Supplementary Note 1. Net accumulation rate of phytoplankton biomass is described in Supplementary Note 2 and the zonal-mean iron budget in Supplementary Note 3. Supplementary Note 4 gives details on the eddy parametrization configurations and Supplementary Note 5 describes the iron flux associated with the mixed-layer instability parametrization. Time-depth plots of iron concentration for each run are given in Supplementary Note 6. Supplementary Note 7 gives the resolution dependence of eddy subduction of phytoplankton.

Supplementary Figure 1. Zonal-mean iron budget (eqn. (S5)) from the 2km run for annual **a**, austral winter (July, August, September) **b**, spring (October, November, December) **c**, summer (January, February, March) **d**, and autumn mean (April, May, June) **e.** The residual term in grey dashed lines comes from the tracer advective flux scheme which prevents tracer concentrations from taking negative values.

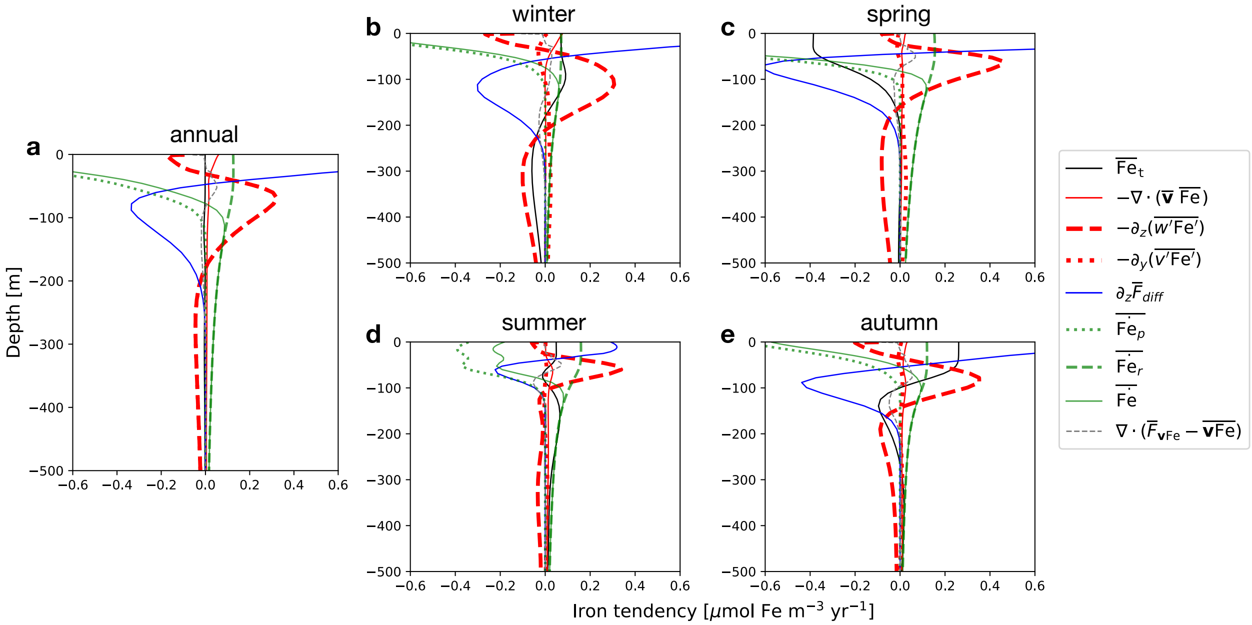

Supplementary Figure 2. Time-depth plots of primary production (PP) in [mgC Fe m^{-3} d^{-1}] **a**, iron consumption by biology ($\dot{Fe}_p$) **b** and source by remineralization ($\dot{Fe}_r$) **c** in [μmol Fe m^{-3} yr^{-1}] from the 2km run (Supplementary Note 2).

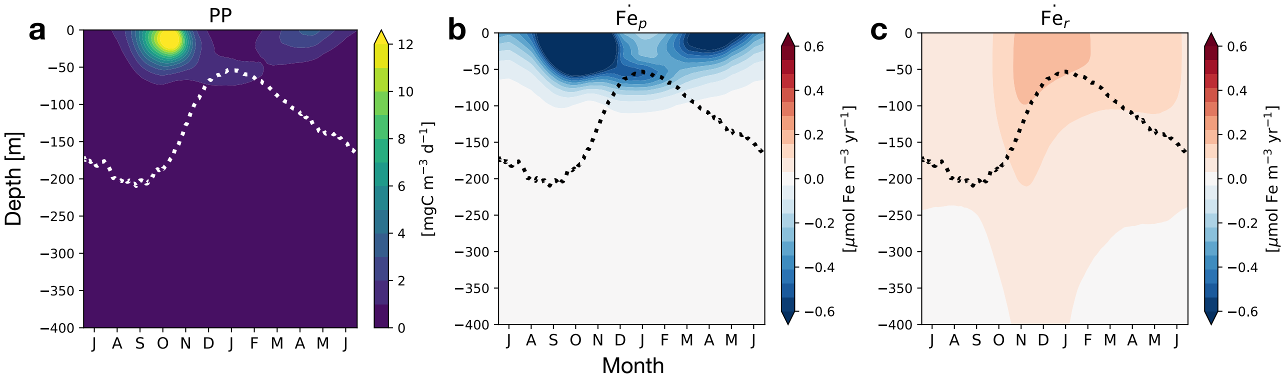

Supplementary Figure 3. Panels **a** and **c** are the same as in Fig. 2 in the main text. **b** Primary production rates in green and the net accumulation, division and loss rates (Supplementary Note 3). Vertically integrated zooplankton biomass is plotted using monthly-averaged outputs.

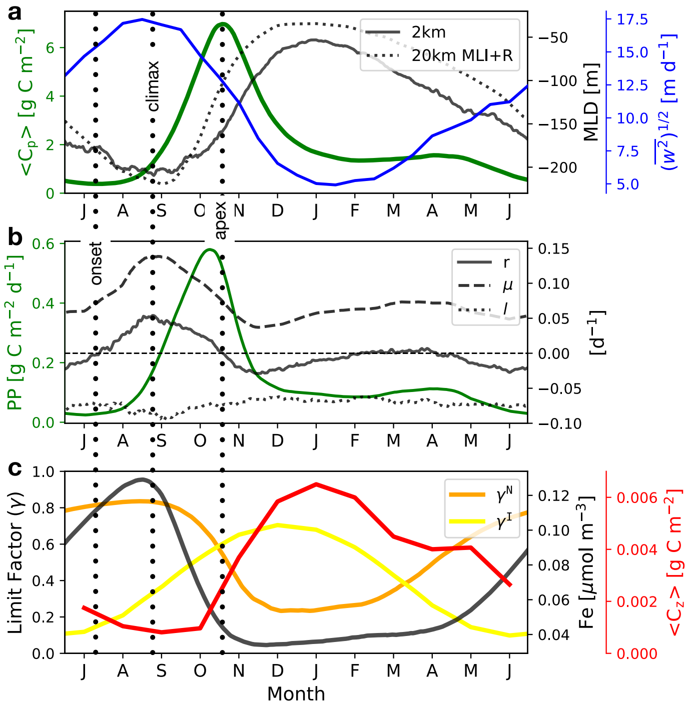

Supplementary Figure 4. Contours of iron concentration are shown in color and isotherms (˚C) in black-and-white shaded contours for the 2km run **a**, 5km run **b**, 20km MLI+R run **c**, and 100km GM+R run **d** (Supplementary Note 4).

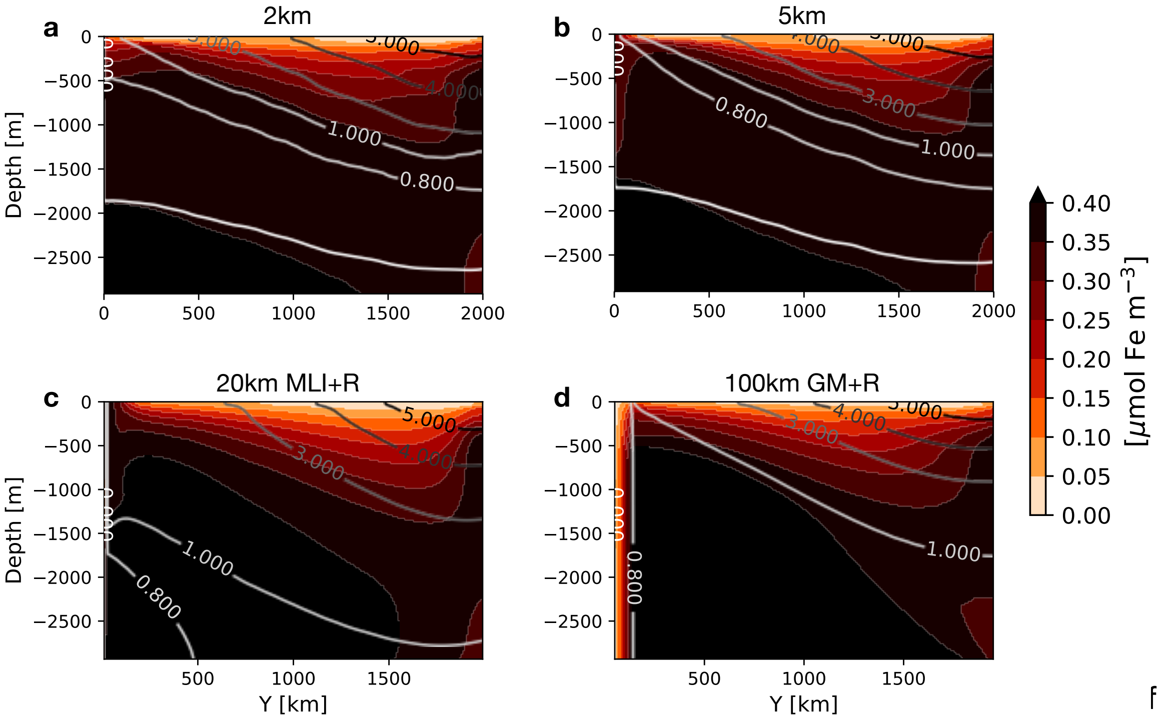

Supplementary Figure 5. Time-depth plots of the vertical eddy iron flux in [μmol Fe m^{-2} yr^{-1}] for runs without any eddy parametrization are shown for each resolution **a-c**.

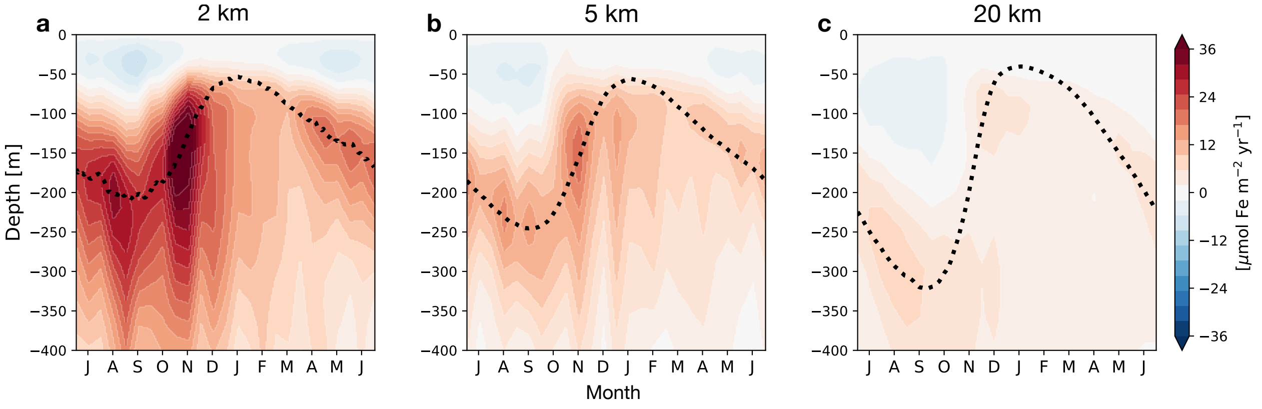

Supplementary Figure 6. Time-depth plots of the vertical total (KPP+Redi+MLI+"resolved" eddy) flux **a** is shown along with the parametrized MLI contribution **b** and resolved eddy contribution **c** of iron in [μmol Fe m^{-2} yr^{-1}] from the 20km MLI+R run (Supplementary Note 5). Note the change in the range of the colorbars.

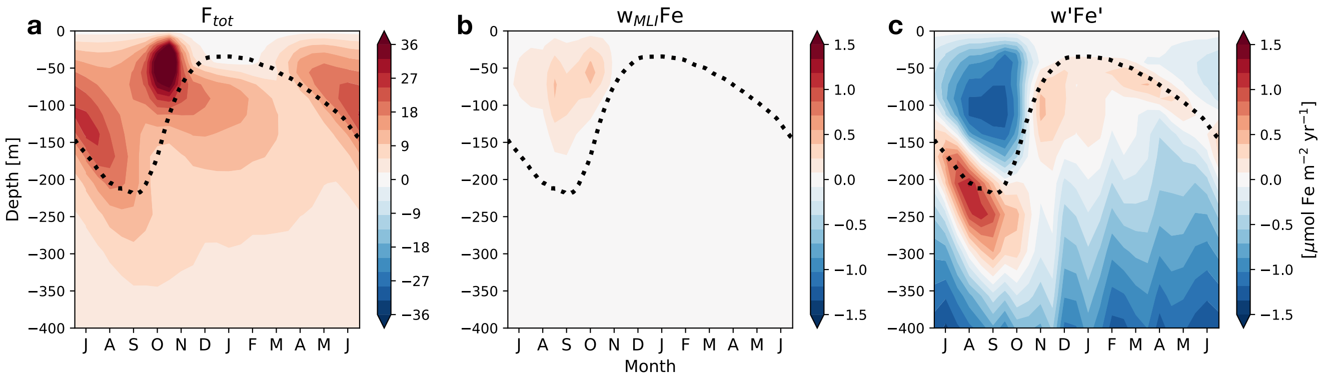

Supplementary Figure 7. Time-depth plots of iron concentration in [μmol Fe m^{-3}] for the 5km **a**, 20km MLI+R **b** and 100km GM+R run **c** (Supplementary Note 6).

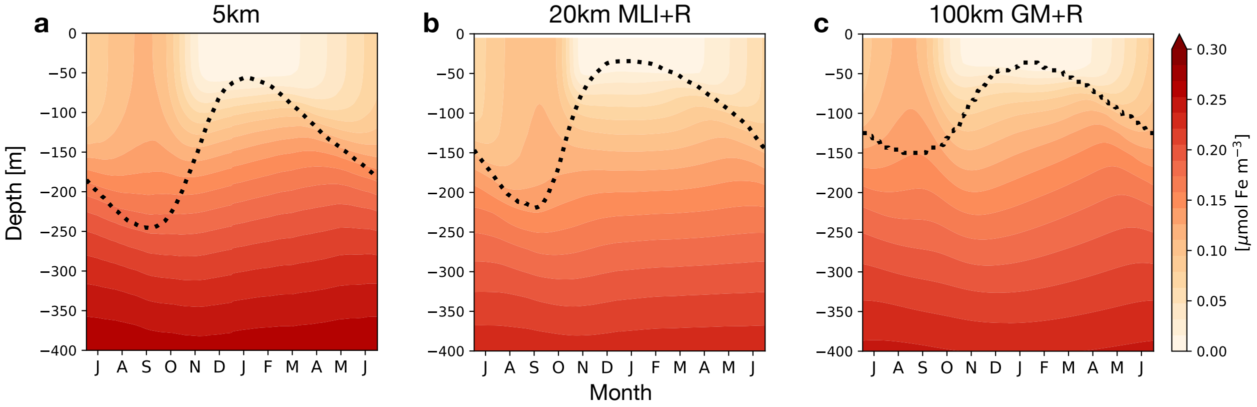

Supplementary Figure 8. Time-depth plots of eddy phytoplankton transport ($w'C_p'$ [mg C m^{-2} yr^{-1}]) for the 2km run (Supplementary Note 7) **a.** The black dotted line shows the the MLD. **b** Time series of the vertically integrated primary production rate ($\langle$PP$\rangle$) and $w'C_p'$ for each run without eddy parametrizations at the ML base or 100m depth whichever is deeper. The 2km run is shown in solid, 5km run in dashed and 20km in dotted lines.

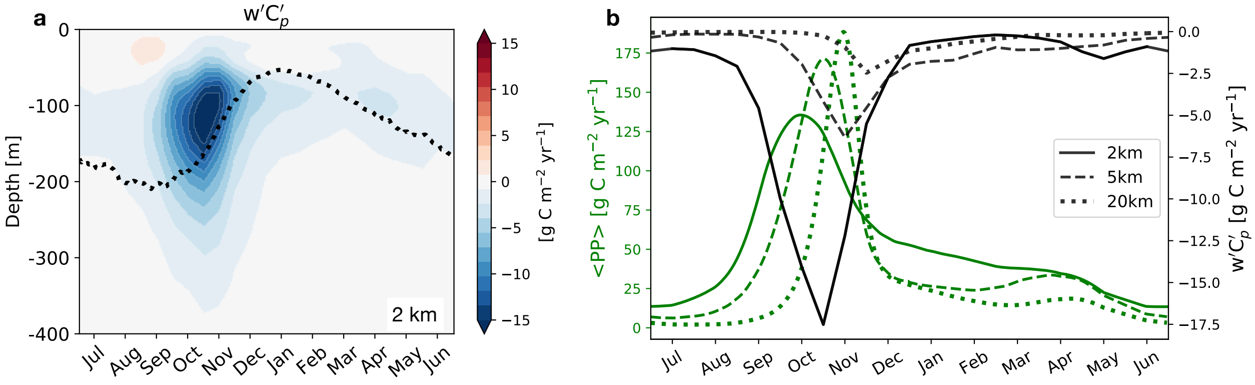

Supplementary Table 1. Physical parameter values for each resolution. The piston velocity is defined as the top grid cell height divided by the relaxation time scale. The parameters with (*) are only used for the 20 km run with MLI parametrization turned on[13]. Further information on other variables is shown in Balwada's Supplementary Material Table 1[1].

Parameter	Value	Units
Horizontal resolution	100, 20, 5, 2	km
Time step	4800, 1800, 150, 40	s
Spin up	1315, 200, 35 (from 20 km), 4.5 (from 5 km)	years
SST relax piston velocity	1/3	m day^{-1}
*Typical width of ML fronts	2	km
*MLI efficiency	0.07	–
*MLI time scale	2×10^{-6} (5.8)	s^{-1} (day)
*Maximum grid-scale	110	km
GM Visbeck coefficient	0.015	–
GM Visbeck length	200	km
GM Visbeck depth	1000	m

Supplementary Table 2. Darwin parameter values used in our configuration where $i\ (=1,2)$ corresponds to the large (diatoms which have an additional silicate dependence) and small phytoplankton species respectively. In Dutkiewicz[2], the units of the half-saturation constants are in [μM] (micromole per liter), equivalent to [mmole m $^{-3}$].

Parameter	Symbol	Value	Units
Phytoplankton max. growth rates	$\mu_{\text{max}_{(1,2)}}$	1/1.24, 1/1.8	days^{-1}
Mortality rates	$m_{P_{(1,2)}}$	15, 12	days
	$m_{Z_{(1,2)}}$	40, 40	days
Zooplankton max. grazing rates	g_{max_a}	2.8	days
	g_{max_b}	16.8	days
Half-saturation constants	$\kappa_{\text{PO}_{4(1,2)}}$	0.035, 0.015	mmole m^{-3}
	$\kappa_{\text{NO}_{3(i)}}$	$\kappa_{\text{PO}_{4(i)}} \times r_{\text{N:P}}$	mmole m^{-3}
	$\kappa_{\text{Fe}_{T(i)}}$	$\kappa_{\text{PO}_{4(i)}} \times r_{\text{Fe:P}}$	mmole m^{-3}
	$\kappa_{\text{Si}_{(1)}}$	$\kappa_{\text{PO}_{4(1)}} \times r_{\text{Si:P}}$	mmole m^{-3}
Phytoplankton elemental ratios	$r_{\text{N:P}}$	16	–
	$r_{\text{Si:P}}$	16	–
	$r_{\text{Fe:P}}$	10^{-3}	–
PAR saturation constants	$\kappa_{\text{par}_{(1,2)}}$	0.018, 0.01	(μEin m^{-2} s^{-1})$^{-1}$
PAR inhibition constants	$\kappa_{\text{inh}_{(1,2)}}$	1.05×10^{-3}, 5.9×10^{-3}	(μEin m^{-2} s^{-1})$^{-1}$
Normalization constant	T_0	0.589	–
Activation temperature	T_{Ae}	4040	K
Absolute zero temperature	T_{kel}	273.15	K
Reference temperature	T_{ref}	277.15	K
Nutrition relaxation time scale	–	30	days

Supplementary Note 1.

Physical configuration

We use the hydrostatic configuration of the Massachusetts Institute of Technology general circulation model[3] (MITgcm). The channel domain ($L_\mathrm{x} = 1000$ km $\times$ $L_\mathrm{y} = 2000$ km $\times$ $H = 2985$ m) is flat bottom and zonally re-entrant on a β-plane centered around 49S ($f_0 = -1.1 \times 10^{-4}$ s^{-1}, $\beta = 1.4 \times 10^{-11}$ m^{-1} s^{-1}). The horizontal grids are on a Cartesian coordinate and the runs with 100 km and 20 km resolution have 40 vertical levels with 10 m near the surface, and the 5 km and 2 km runs have 76 vertical levels with 1 m near the surface. Monthly varying sea-surface temperature (SST) relaxation and zonal wind stress are applied at the surface; SST increases from 0°C to 8°C from south to north, and the zonal cosine-squared-shaped wind stress takes its maximum amplitudes between 0.1-0.2 N m^{-2} at the center of the meridional extent and is tapered to zero at the northern and southern 50km extent of the domain. The Leith-scheme horizontal[4] and vertical viscosity values of $A_\mathrm{h} = 2.15$ m^2 s^{-1} and $A_\mathrm{v} = 5.6614 \times 10^{-4}$ m^2 s^{-1} are used. We apply no-slip boundary conditions at the channel walls and bottom with the latter having a quadratic drag, $C_\mathrm{d} = 2.1 \times 10^{-3}$. Other parameter values are given in Supplementary Table 1.

Biogeochemical configuration

We adopt a two-species ecosystem model, simplified from the Darwin biogeochemical model[5], used in previous global biogeochemical studies[2,6]. The model considers the full biogeochemical cycle of oxygen, carbon, phosphate, nitrate, silicate and for the interest of our study, iron with each tracer existing in organic and inorganic form and nutrient relaxation is applied at the northern boundary. The relaxation profiles for PO_4, NO_3 and SiO_2 were taken from the World Ocean Atlas at 45S and interpolated onto our model vertical grid. We use the monthly climatological products down to 500~m where monthly data is available and append the annual climatology below. Monthly iron profiles were taken from the Biological Southern Ocean State Estimate[7] (BSOSE) as the Geotraces dataset[8] did not have sufficient temporal and spatial resolution. In an effort to compensate for the lack of dust, glacial and bathymetric sources, we chose 50S of BSOSE which had higher concentrations than at 45S but details of the relaxation profiles ultimately did not make a difference in surface concentrations as the spun up interior iron concentration was rather insensitive to the details of the relaxation profile (not shown). Photosynthetically available radiation is prescribed at the surface as a meridional linear fit to the monthly and zonal mean of SeaWiFS product between latitudes of 45-60S and takes its minimum in June and maximum in December. For completeness, we briefly describe the tracer equations for phytoplankton species i and iron,

$$\frac{\mathrm{DP}_i}{\mathrm{D}t} = \mu_i \mathrm{P}_i - m_i^{\mathrm{P}} \mathrm{P}_i - G_i + D_i \quad \text{(S1)}$$

$$\frac{\mathrm{DFe}}{\mathrm{D}t} = -\sum_i \mu_i \mathrm{P}_i r_{\mathrm{Fe:P}} + S_{\mathrm{Fe}} + D_{\mathrm{Fe}} \quad \text{(S2)}$$

where the source term (S_{Fe}) is due to remineralization and the northern boundary relaxation in a domain-wide sense. The biogeochemical sink due to primary production contribution was derived offline as $\dot{\mathrm{Fe}}_p = -\,\mathrm{PP} \times r_{\mathrm{Fe:P}}$ and remineralization as the difference from the net biogeochemical source term, i.e. $\dot{\mathrm{Fe}}_r = \dot{\mathrm{Fe}} - \dot{\mathrm{Fe}}_p$, using daily-averaged outputs. Details of mortality (m), grazing (G) and diffusion (D) terms will be left to Dutkiewicz[2]. From eqn. (S1), we see that net primary production is defined as $\mathrm{PP} = \sum_i \mu_i \mathrm{P}_i$. Light, temperature and nutrient modification to the phytoplankton growth rate are implemented as

$$\mu_i = \mu_{\mathrm{max}_i} \gamma_i^{\mathrm{I}} \gamma_i^{\mathrm{T}} \gamma_i^{\mathrm{N}}$$

where μ_{max_i}, γ_i^{I}, γ_i^{T} and γ_i^{N} are the maximum growth rate of phytoplankton i and limitation factors by temperature, light and nutrients respectively[2]. Light limitation is calculated as

$$\gamma_i^{\mathrm{I}} = \min\left[F_0^{-1}\left(1 - \exp[-\kappa_{\mathrm{par}}\mathrm{PAR}]\right)\exp[-\kappa_{\mathrm{inh}}\mathrm{PAR}],1\right] \quad \text{for PAR} > 1 \quad \text{(S3)}$$

and is 0 for PAR < 1 where PAR is the photosynthetically available radiation in units of $[\mu\mathrm{Ein\ m^{-2}s^{-1}}]$ and

$$F_0 = \frac{\kappa_{\mathrm{par}}}{\kappa_{\mathrm{par}} + \kappa_{\mathrm{inh}}} \exp\left[\frac{\kappa_{\mathrm{inh}}}{\kappa_{\mathrm{par}}} \log\left[\frac{\kappa_{\mathrm{inh}}}{\kappa_{\mathrm{par}} + \kappa_{\mathrm{inh}}}\right]\right].$$

Our ecosystem is iron limited year round so the nutrient factor becomes

$$\gamma_i^{\mathrm{N}} = \frac{\mathrm{Fe}}{\mathrm{Fe} + \kappa_{\mathrm{Fe}_i}} \quad \text{(S4)}$$

where κ_{Fe_i} is the half-saturation constant of iron for phytoplankton i. Further details on the biogeochemical parameters are shown in Supplementary Table 2.

Supplementary Note 2.

Due to the zonally-reentrant configuration of our simulation, it is natural to consider zonal-mean quantities. Based on eqn. (S2), the zonal-seasonal mean iron budget shown in Supplementary Fig. 1 becomes

$$\overline{\frac{\partial \mathrm{Fe}}{\partial t}} = -\,\nabla \cdot \overline{\mathbf{v}}\overline{\mathrm{Fe}} - \overline{\frac{\partial}{\partial y}(v'\mathrm{Fe}')} - \overline{\frac{\partial}{\partial z}(w'\mathrm{Fe}')} + \overline{\dot{\mathrm{Fe}}} + \overline{D_{\mathrm{Fe}}} \quad \text{(S5)}$$

where the biogeochemical source/sink terms are lumped together as $\dot{\mathrm{Fe}}$ and $\overline{(\,\cdot\,)}$ indicates the zonal-seasonal mean over the meridional extent of $y = 600\text{-}1400$ km. We have no contribution from zonal advection due to the zonally re-entrant configuration, viz. $\overline{\partial_x(u\mathrm{Fe})} = 0$. As was shown in Fig. 3 in the main text, the vertical eddy transport reaches below the MLD into the water column to bring up iron indicated by positive values ($-\partial_z\overline{(w'\mathrm{Fe}')} > 0$; red dashed) and passes it on to the diffusive flux ($\partial_z \overline{F}_{\mathrm{diff}}$; blue), in our case due to KPP mixing. The net biogeochemical source/sink term ($\overline{\dot{\mathrm{Fe}}}$) is a net sink near the surface year round due to primary production ($\overline{\dot{\mathrm{Fe}}_p}$) overwhelming the source by remineralization ($\overline{\dot{\mathrm{Fe}}_r}$; green dashed). This is also shown in

Supplementary Fig. 2 as time-depth plots where the relation $|\overline{\dot{\text{Fe}}_p}| > |\overline{\dot{\text{Fe}}_r}|$ holds year round in the upper 100 m. Associated with the spring and autumn blooms are maxima in the iron consumption ($\overline{\dot{\text{Fe}}_p}$) which mirrors primary production (PP). The contribution due to horizontal eddy transport ($-\partial_y(\overline{v'\text{Fe}'})$; red dotted) and mean advection ($-\nabla \cdot (\overline{\mathbf{v}}\overline{\text{Fe}})$; red solid) is small compared to the other terms in our simulation. Although our wintertime biogeochemical consumption of iron is within the bounds of observations, it is too low during summer; estimates based on ship-track measurements[9,10] tend to be on the order of 100 μmol m^2 yr^{-1} while as it is roughly 35 μmol m^2 yr^{-1} integrating over the top 100 m in our model (Supplementary Fig. 1**d**; green dotted line). Due to the lack of pelagic community transition, our spring bloom ebbs too soon and is insufficiently sustained over the summer.

Supplementary Note 3.
In addition to the onset and apex, the climax can be defined via the net accumulation rate ($r = \frac{d}{dt} \ln\langle \text{C}_\text{p} \rangle$)[11] as a maximum in r (Supplementary Fig. 3**b**). Along with the accumulation rate, we show the vertically integrated primary production rate ($\langle \text{PP} \rangle$), net population division rate ($\mu = \frac{\langle \text{PP} \rangle}{\langle \text{C}_\text{p} \rangle}$) and loss rate ($l = r - \mu$) — the net effect of phytoplankton mortality and grazing by zooplankton. The loss rate is relatively constant year round and if anything its amplitude decreases when zooplankton ($\langle \text{C}_\text{Z} \rangle$) is increasing (Supplementary Fig. 3**b**,**c**) implying a bottom-up (iron limited) regulation of the spring bloom.

Supplementary Note 4.
For the 100 km GM+R run, we did not have a case with the MLI parametrization[12] turned on due to implementation conflicts with the tapering scheme within MITgcm; the overturning streamfunction of MLI was implemented in a similar fashion to GM in *advective* form, while as Ferrari's tapering scheme[13] required GM to be in *skew flux* form[14]. The MLI parametrization was originally developed to represent the density restratification due baroclinic instability within the mixed layer and not submesoscale tracer transport[12]. As we show in the 20 km resolution runs, the parametrization does not enhance cross-ML-base tracer transport (Supplementary Fig. 5**b**) and hence, should not qualitative affect our results at 100 km resolution. One final caveat is that, although we tested 100 km resolution runs with a constant GM coefficient (K_GM), in order to get the best agreement in stratification with the 2 km run, allowing for K_GM to have meridional dependence[15] and proper tapering near the boundaries[13] were both needed. This is consistent with previous studies in the Southern Ocean that diagnose K_GM[16] and look at GM's response to changes in the surface wind forcing[17]. The Redi diffusivity of 200 m^2 s^{-2} in the 20 km MLI+R run was chosen to be on the same order as used in non-eddying general circulation models[18] but smaller due to our resolution

being mesoscale permitting. Since the Redi diffusivity is a free parameter in our case, we could increase it to have more isopycnal eddy iron transport. Considering the insufficient restratification (Supplementary Fig. 4), however, we argue that it would be getting the right amount of eddy transport for the wrong reasons. We also experimented with the GM parametrization at 20 km resolution, but GM's main effect was to damp the resolved eddy field[19]. Applying GM at 20 km resolution steepened rather than slumping the isopycnals. The configuration with Redi but not GM enhanced the isopycnal diffusion of tracers without suppressing the resolved eddies.

Supplementary Note 5.

We show the total vertical flux from the 20km MLI+R (Supplementary Fig. 6**a**) and the contribution from the MLI parametrization (Supplementary Fig. 6**b**) on iron transport. The total flux is the sum of the diffusive (KPP+Redi) flux, parametrized MLI and resolved eddy flux. The cross-ML-base iron flux in Supplementary Fig. 6**a** is dominated by the Redi contribution and KPP mixing within the ML with the contribution from MLI being negligible.

Supplementary Note 6.

Supplementary Fig. 7 shows the time-depth plots of iron stratification in the 5km, 20km MLI+R and 100km GM+R runs. The stratification is the weakest for the 20km MLI+R run in the top 200m.

Supplementary Note 7.

Supplementary Fig. 8**a** shows the time-depth plots of eddy phytoplankton transport ($w'\mathrm{C}_\mathrm{p}'$) for the 2km run. The magnitude of $w'\mathrm{C}_\mathrm{p}'$ increases with resolution (Supplementary Fig. 8**b**). The eddy terms are defined as the residual of subtracting out the seasonal zonal mean from snapshot outputs every 15 days. The spring primary production peaks earlier and the baseline productivity increases for higher resolution runs because there's more iron available due to eddy supply. The annual maximum of eddy phytoplankton subduction occurs slightly after primary production takes its annual maximum.

Supplementary References

1. Balwada, D., Smith, K. S. & Abernathey, R. Submesoscale vertical velocities enhance tracer subduction in an idealized antarctic circumpolar current. *Geophysical Research Letters* **45**, 9790—9802 (2018).
2. Dutkiewicz, S., Follows, M. J. & Braggs, J. G. Modeling the coupling of ocean ecology and biogeochemistry. *Global Biogeochemical Cycles* **23**, GB4017 (2009).
3. Marshall, J., Adcroft, A., Hill, C., Perelman, L. & Heisey, C. A finite-volume, incompressible Navier-Stokes model for studies of the ocean on parallel computers. *Journal of Geophysical Research: Oceans* **102**, 5753—5766 (1997).

4. Bachman, S. D., Fox-Kemper, B. & Pearson, B. A scale-aware subgrid model for quasi-geostrophic turbulence. *Journal of Geophysical Research: Oceans* **122**, 1529—1554 (2017).
5. Follows, M. J., Dutkiewicz, S., Grant, S. & Chisholm, S. W. Emergent biogeography of microbial communities in a model ocean. *Science* **315 (5820)**, 1843—1846 (2007).
6. Gloege, L., McKinley, G. A., Mouw, C. B. & Ciochetto, A. B. Global evaluation of particulate organic carbon flux parametrization and implications for atmospheric pCO_2. *Global Biogeochemical Cycles* **31**, 1192—1215 (2017).
7. Verdy, A. & Mazloff, M. A data assimilating model for estimating southern ocean biogeochemistry. *Journal of Geophysical Research: Oceans* **122**, 6968—6988 (2017).
8. Tagliabue, A. *et al*. Surface-water iron supplies in the southern ocean sustained by deep winter mixing. *Nature Geosciences* **7**, 314—320 (2014).
9. Ellwood, M. J., Boyd, P. W. & Sutton, P. Winter-time dissolved iron and nutrient distributions in the subantarctic zone from 40-52S. *Geophysical Research Letters* **35 (11)**, 155—160e (2008).
10. Bowie, A. *et al*. Biogeochemical iron budgets of the Southern Ocean south of Australia: Decoupling of iron and nutrient cycles in the subantarctic zone by the summertime supply. *Global Biogeochemical Cycles* **23 (4)**, GB4043 (2009).
11. Behrenfeld, M. J. & Boss E. S. Student's tutorial on bloom hypotheses in the context of phytoplankton annual cycles. *Global Change Biology* **24**, 55—77 (2018).
12. Fox-Kemper, B. *et al*. Parametrization of mixed layer eddies. iii: Implementation and impact in global ocean climate simulations. *Ocean Modeling* **39**, 61—78 (2011).
13. Ferrari, R. McWilliams, J. C., Canuto, V. M & Dubovikov, M. Parametrization of eddy fluxes near oceanic boundaries. *Journal of Climate* **21**, 2770—2789 (2008).
14. Griffies, S. M. The Gent-McWilliams skew flux. *Journal of Physical Oceanography* **28**, 831—841 (1998).
15. Visbeck, M., Marshall, J., Haine, T. & Spall, M. Specification of eddy transfer coefficients in coarse-resolution ocean circulation models. *Journal of Physical Oceanography* **27**, 381—402 (1997).
16. Abernathey, R., Ferriera, D. & Klocker, A. Diagnostics of isopycnal mixing in a circumpolar channel. *Ocean Modelling* **72**, 1—16 (2013).
17. Gent, P. R. & Dabanasoglu, G. Response to increasing southern hemisphere winds in CCSM4. *Journal of Climate* **24**, 4992—4998 (2011).
18. Gnanadesikan, A., Pradal, M.-J. & Abernathey, R. Isopycnal mixing by mesoscale eddies significantly impacts oceanic anthropogenic carbon uptake. *Geophysical Research Letters* **42**, 4249—4255 (2015).
19. Hallberg, R. Using a resolution function to regulate parametrizations of oceanic mesoscale eddy effects. *Ocean Modelling* **72**, 92—103 (2013).
