# Supplementary material for: Vertical eddy iron fluxes support primary production in the open Southern Ocean
Source: Nat Commun. 2020 Feb 28;11:1125. doi: 10.1038/s41467-020-14955-0 (PMC7048949; doi:10.1038/s41467-020-14955-0)
Supplement: Supplementary file 3 — Reporting Summary [file 41467_2020_14955_MOESM3_ESM.pdf]

nature research

Corresponding author(s): Takaya Uchida

Last updated by author(s): Oct 23, 2019

Reporting Summary

Nature Research wishes to improve the reproducibility of the work that we publish. This form provides structure for consistency and transparency in reporting. For further information on Nature Research policies, see Authors & Referees and the Editorial Policy Checklist .

Statistics

For all statistical analyses, confirm that the following items are present in the figure legend, table legend, main text, or Methods section.

n/a	Confirmed	
☒	☐	The exact sample size (*n*) for each experimental group/condition, given as a discrete number and unit of measurement
☒	☐	A statement on whether measurements were taken from distinct samples or whether the same sample was measured repeatedly
☒	☐	The statistical test(s) used AND whether they are one- or two-sided<br>*Only common tests should be described solely by name; describe more complex techniques in the Methods section.*
☒	☐	A description of all covariates tested
☒	☐	A description of any assumptions or corrections, such as tests of normality and adjustment for multiple comparisons
☒	☐	A full description of the statistical parameters including central tendency (e.g. means) or other basic estimates (e.g. regression coefficient) AND variation (e.g. standard deviation) or associated estimates of uncertainty (e.g. confidence intervals)
☒	☐	For null hypothesis testing, the test statistic (e.g. *F*, *t*, *r*) with confidence intervals, effect sizes, degrees of freedom and *P* value noted<br>*Give P values as exact values whenever suitable.*
☒	☐	For Bayesian analysis, information on the choice of priors and Markov chain Monte Carlo settings
☒	☐	For hierarchical and complex designs, identification of the appropriate level for tests and full reporting of outcomes
☒	☐	Estimates of effect sizes (e.g. Cohen's *d*, Pearson's *r*), indicating how they were calculated

Our web collection on statistics for biologists contains articles on many of the points above.

Software and code

Policy information about availability of computer code

Data collection	Version "checkpoint63n" of the Massachusetts Institute of Technology general circulation model (MITgcm) was used to run our simulations.
Data analysis	Open source Python packages of "xgcm 0.2.0" and "xmitgcm 0.4.1" were used to analyze the simulation outputs.

For manuscripts utilizing custom algorithms or software that are central to the research but not yet described in published literature, software must be made available to editors/reviewers. We strongly encourage code deposition in a community repository (e.g. GitHub). See the Nature Research guidelines for submitting code & software for further information.

Data

Policy information about availability of data

All manuscripts must include a data availability statement. This statement should provide the following information, where applicable:

- Accession codes, unique identifiers, or web links for publicly available datasets
- A list of figures that have associated raw data
- A description of any restrictions on data availability

The model configuration is available on Github (doi:10.5281/zenodo.3266400) and simulation outputs for 15-daily snapshot and monthly-averaged outputs of physical variables are available on Pangeo (doi:10.5281/zenodo.3358021).

Field-specific reporting

Please select the one below that is the best fit for your research. If you are not sure, read the appropriate sections before making your selection.

Ecological, evolutionary & environmental sciences study design

All studies must disclose on these points even when the disclosure is negative.

Study description	We examine the open Southern Ocean ecosystem by running a physical/biogeochemical numerical simulation. The focus of our study is to quantify the relative role of eddy iron transport in the iron budget and its impact on primary production in the open Southern Ocean.
Research sample	N/A
Sampling strategy	N/A
Data collection	Data were generated via computer simulations using the MITgcm run on the Habanero supercomputing system of Columbia University in the city of New York.
Timing and spatial scale	Due to limitations in data storage capacity, we have one year of data outputs for the 2km resolution run. We have five years of simulation output for the 5km resolution run and 10 years for the 20km resolution run respectively.
Data exclusions	Data during the spin-up period of the simulation were excluded from our analysis.
Reproducibility	The model configuration is available on Github (doi:10.5281/zenodo.3266400).
Randomization	N/A
Blinding	As our study is a modelling one, blinding was not relevant.

Did the study involve field work? ☐ Yes ☒ No

Reporting for specific materials, systems and methods

We require information from authors about some types of materials, experimental systems and methods used in many studies. Here, indicate whether each material, system or method listed is relevant to your study. If you are not sure if a list item applies to your research, read the appropriate section before selecting a response.

Materials & experimental systems

n/a	Involved in the study
☒	☐ Antibodies
☒	☐ Eukaryotic cell lines
☒	☐ Palaeontology
☒	☐ Animals and other organisms
☒	☐ Human research participants
☒	☐ Clinical data

Methods

n/a	Involved in the study
☒	☐ ChIP-seq
☒	☐ Flow cytometry
☒	☐ MRI-based neuroimaging
